# Quality of Residual Neuromuscular Control and Functional Deficits in Patients with Spinal Cord Injury

**DOI:** 10.3389/fneur.2013.00174

**Published:** 2013-11-07

**Authors:** Alexander V. Ovechkin, Todd W. Vitaz, Daniela G. L. Terson de Paleville, William B. McKay

**Affiliations:** ^1^Department of Neurological Surgery, University of Louisville, Louisville, KY, USA; ^2^Norton Neuroscience Institute, Louisville, KY, USA; ^3^Exercise Physiology, University of Louisville, Louisville, KY, USA; ^4^Hulse Spinal Cord Injury Laboratory, Shepherd Center, Atlanta, GA, USA

**Keywords:** spinal cord injury, motor control, assessment, functional outcome, neurological outcome, FIM, SCIM, WISCI

## Abstract

**Study Design:** Prospective cohort study.

**Objective:** This study examined the relationship between motor control and clinical function outcomes after spinal cord injury (SCI).

**Setting:** University of Louisville, Louisville, KY, USA.

**Materials:** Eleven persons with SCI and 5 non-injured subjects were included in this study.

**Methods:** The ASIA Impairment Scale (AIS) was used to categorize injury level and severity. Multi-muscle, surface EMG (sEMG) recording, was carried out using a protocol of reflex and volitional motor tasks and was analyzed using a vector-based tool that calculates index values that relate a distribution of multi-muscle activation pattern of each SCI subject to the prototype obtained from non-injured subject group and presents overall magnitude as a separate value. Functional Independence Measure motor sub-scale, Spinal Cord Injury Independence Measure (SCIM-III), and Walking Index for Spinal Cord Injury (WISCI) scale scores were compared to neurophysiological parameters.

**Results:** AIS category and injury level correlated significantly with the WISCI and SCIM mobility sub-scales. sEMG-derived parameters were significantly correlated with SCIM and WISCI scores but only for examinations carried out 48 or more days post-injury.

**Conclusion:** These results supported the hypothesis that clinically relevant function after SCI is related to the degree to which functional organization within the central nervous system is disrupted. Further, due likely to the constraints placed on the expression of functional ability by early post-injury immobilization and hospitalization, neurophysiological assessment of motor function may provide better sensitivity and reliability than can be obtained using the clinical function scales examined here within the early period after injury.

## Introduction

During the past two decades, spinal cord injury (SCI) has seen significant advancement in the areas of neuroprotection, neural repair and regeneration, medical and surgical management, and rehabilitation. However, the development and implementation of outcome scales to adequately assess these new strategies have lagged.

The overall goal of all of these treatments and interventions is the improvement or restoration of function. However, the best ways to measure and assess these changes remain controversial. The World Health Organization has developed the International Classification of Function (ICF) which is a biopsychosocial schema that evaluates the impact of disease on function and quality of life. The ICF is comprised of three domains: (1) body function and structure (physiological functions and anatomical body parts), (2) activities (execution of a task), and (3) participation (involvement in life situations) (1, 2). As one moves across these domains to assess quality of life, the complexity of measuring the impact of a given therapeutic intervention increases because of the increased impact of environmental and personal factors (2). Currently most outcome scales measure “clinically meaningful” change in the activity and participation domains. Therefore the benefit brought by an experimental intervention which improves function within spinal cord neural circuitry may be “lost in the noise” if the outcome measure used assesses activity or participation domains as its endpoint (2).

The classification of SCI developed by the American Spinal Injury Association (ASIA) is currently the assessment tool used to evaluate the body function and structure domain (3). This ASIA impairment scale (AIS) has been shown to be a reliable tool in the assessment and characterization of injury level and lesion severity (4, 5). However, one of the greatest criticisms of this assessment tool is the subjectivity of the sensory and motor testing and the low inter-observer reliability unless the raters have received extensive training and have vast experience with this tool.

As a result, work has been done to develop other assessment tools to assess in the body function and structures domain. One such tool developed over the past decade has been functional electromyography (fEMG). fEMG incorporates the recording of spinal motor output in the form of pooled motor unit firing recorded from multiple muscles during the performance of selected reflex and volitional motor tasks (6). Similar to the AIS, this neurophysiological tool measures the ability of the patient to volitionally activate a specific muscle, however fEMG has the increased sensitivity to assess the effects of complex multi-muscle organization and interaction (agonist activation and antagonist inhibition) that is required to perform these tasks (7). In addition, quantification of the distribution of spinal motor output across multiple muscles is recorded in an objective process that eliminates the issues surrounding intra- and inter-rater reliability (7). Test-retest reliability for the calculated parameters was examined and found to be quite high (8). Therefore, such neurophysiological assessment promises improved reliability and adds a spatial distribution domain to the information currently offered by the AIS in the assessment of motor control for use in the treatment of SCI. This study sought to examine the relationship between such fEMG-quantified neural circuit function and currently applied measures that assess within the activities domain of the ICF in an effort to expand the arsenal of tools available for use in evaluating SCI treatment.

## Materials and Methods

Participants were enrolled after informed consent was obtained as approved by the Institutional Review Board for Human Studies of the University of Louisville. Function scale scores and neurophysiological assessment were carried out within 1 day of each other in 13 sessions from 11 subjects with SCI. Three were female and ages ranged from 23 to 82 (48 ± 19; average ± SD) years. Follow-up sessions were obtained in two. SCI injury levels ranged from C2 to S1 and time of study was 3 days to 14 (7.9 ± 17) months post-injury (Table 1). AIS examination was carried out within the same day for seven of the sessions. For the other six sessions, AIS was assessed between 2 and 44 (6.4 + 12.5) days before. Five subjects were motor complete, AIS-A, and eight were motor-incomplete, one AIS-C, and seven AIS-D. In addition to the SCI subjects, five otherwise matched non-injured healthy subjects underwent the brain motor control assessment (BMCA) recording to provide normative reference data. One was female and their ages ranged from 20 to 59 (39 ± 18) years of age.

**Table 1:** **Thirteen measurement sessions carried out in 11 subjects, with follow-up (f) examinations in 2**.

Subject number	AIS	Injury level	Time pass onset	FIM motor	WISCI	SCIM-III			Similarity index			Magnitude		
						Self care	Mobility	Total	Mean upper SI	Mean lower SI	Mean upper and lower SI	Mean upper Mag	Mean lower Mag	Mean upper and lower Mag
1	A	T8	9 days	18	0	4	0	14	NT	0.00	NT	NT	0	NT
2	A	C8	19 days	16	0	6	2	25	NT	0.00	NT	NT	0	NT
3	D	C8	3 days	69	8	13	25	77	0.57	0.88	0.72	47	57	52
4	D	C6	4 days	24	8	12	18	70	0.84	0.94	0.89	48	137	93
5	D	C6	5 days	21	0	0	6	36	0.87	0.88	0.88	47	101	74
6	D	C6	10 days (B)	58	17	8	22	67	0.91	0.95	0.93	84	61	73
7	A	T9	4 months	52	0	14	15	59	NT	0.00	NT	NT	0	NT
8	A	C5	5 months	14	0	0	3	13	0.33	0.00	0.17	29	0	15
1f	A	T5	5 months	18	0	4	0	14	NT	0.00	NT	NT	0	NT
9	C	C4	48 days	22	17	6	19	56	0.46	0.89	0.68	24	44	34
10	D	C4	3 months	91	20	19	40	99	0.85	0.88	0.87	48	146	97
9f	D	C2	6 months	87	20	13	24	77	0.61	0.92	0.77	46	80	63
11	D	C2	14 months (B)	78	13	15	25	60	0.80	0.91	0.85	30	61	45

*ASIA impairment scale (AIS) categories for those assessments performed early (1–6) and late (7–11) after injury. Four examinations did not test (NT) upper limbs. Two subjects were taking Baclofen (B) at the time of examination*.

### Assessments

#### Injury level and severity

The American Spinal Injury Association Impairment Scale provides a subjective estimation of voluntary contraction strength for five upper limb (C5 to T1) and five lower limb (L2 to S1) muscles on each side along with the perception of light touch and pin prick for C2 through S5 dermatomes (4).

#### Functional independence measure

For this study, a modified functional independence measure (FIM) was applied which scored only eating, grooming, bathing, dressing upper and lower body, toileting, bladder management, bowel management, transfers (bed/chair/wheelchair, toilet, tub/shower), walking/wheelchair, and walking up stairs. For comparison to the neurophysiological measure of motor control described below, 13 self care, sphincter control, mobility, and locomotion items from the FIM were summed to form a motor score with a maximum potential value of 91 points.

#### Spinal cord independence measure

The spinal cord independence measure (SCIM-III) consists of 17 items from two sub-scales, Self Care and Mobility (9). The most independent persons would have scored 20 in the Self Care sub-scale and 40 for the Mobility sub-scale. The total potential SCIM score of 100 points includes another 40 points from items that make up the Respiration and Sphincter Management sub-scale.

#### Walking index for spinal cord injury

The walking index for spinal cord injury (WISCI) categorizes the ability to ambulate on the basis of a 20-level scale formed from the degree of physical assistance and assistive device use needed to cover a 10-m distance (10, 11).

#### Motor control

The BMCA is a protocol of volitional and reflex motor tasks carried out while recording surface EMG (sEMG) from limb muscles (6). sEMG signals were recorded on a 32-channel AXON Eclipse Neuromonitoring System (AXON Systems, Inc, Hauppauge, NY, USA) with a sampling rate of 2 kHz per channel and a bandpass of 30 Hz to 1 kHz. Recorded muscles included the right and left upper trapezius (UT), biceps brachi (BB), triceps brachi (TB), wrist extensors (WE), and wrist flexors (WF) from the upper limbs. Lower limb muscles recorded included right and left quadriceps (Q), adductor (Add), hamstrings (H), tibialis anterior (TA), and soleus (Sol). The protocol was carried out in the supine position and began with 5 min of relaxation. All volitional tasks were cued with an audible 5-s tone and repeated three times with best relaxation achieved between trials. Subjects were instructed to “move and hold for the duration of the tone” for each phase. This study focused on unilateral voluntary movement of elbow, wrist, hip and knee, and ankle. Data from other segments that included reinforcement maneuvers, passive movement, tendon tap responses, vibration, and plantar withdrawal suppression will not be described but were collected during the standard BMCA recording sessions.

### Data reduction

Surface EMG signals from all recorded muscles were quantified using a root-mean-square (RMS) algorithm that produced values in microvolts per second units for each 5-s analysis window for each of the three trials which were then averaged for each voluntary task. Background noise was measured from a 1-s window prior to the motor task and subtracted from the 5-s window average value for each muscle. Response vectors (RVs) were calculated (7) from: right and left UT, BB, and TB muscles for elbow flexion and extension; right and left UT, BB, TB, WE, and WF muscles for wrist extension; right and left Qd, Add, and H muscles for hip and knee flexion; right and left Qd, Add, H, TA, and Sol muscles for ankle dorsiflexion and plantar flexion. To generate the Prototype Response Vectors (PRVs), the healthy subject RVs were first normalized, then averaged. The PRV was thus comprised of one element for each muscle included for the task, presented in dimensionless units for each task. The Similarity Index is a numerical expression of the similarity of the distribution of sEMG activity in the RV, computed as the (normalized) inner product, or cosine of the solid angle between the vector representing the distribution of activity generated by non-injured subjects, the PRV, and that representing the distribution within the test-subject RV. The similarity index (SI) is thus constrained to lie between −1 and 1. A value of 1.0 for the SI means that the angle was 0 and that the test-subject’s RV had an identical distribution of sEMG activity across muscles as did the non-injured subject group PRV for that task (7, 12–14).

### Data analysis

The relationship between function scale scores and neurophysiological index values was statistically examined for all recordings and then separated into two groups on the basis of when they were recorded: up to 19 days post-injury (early); after 48 days post onset (late) (Table 2). The early set was composed of six studies, two of which were lower limbs only BMCA protocols. The later set was made up of seven studies, two of which were lower limb only protocols. Since the items in FIM and SCIM scales require both upper and lower limb function, their values were compared to neurophysiological parameters derived from the nine studies that examined both upper and lower limbs.

**Table 2:** **Spearman’s rank correlation rho (ρ) values comparing across clinical scale scores**.

Clinical scale collections			*P*
AIS	To	FIM motor	0.57
		WISCI	0.71*
		SCIM self care	0.28
		SCIM mobility	0.76*
		SCIM total	0.72**
Injury level	To	FIM motor	−0.37
		WISCI	−0.74**
		SCIM self care	−0.14
		SCIM mobility	−0.58*
		SCIM total	−0.33
FIM motor	To	WISCI	0.69**
		SCIM self care	0.88**
		SCIM mobility	0.86**
		SCIM total	0.88**
WISCI	To	SCIM self care	0.45
		SCIM mobility	0.84**
		SCIM total	0.74**

*p < 0.05; **p < 0.01.

Similarity index and Mag values from right and left elbow flexion and extension, wrist extension, hip and knee flexion, and ankle dorsiflexion and plantar flexion were averaged together for comparison to FIM and SCIM scores. For the WISCI comparison, right and left lower limb tasks were averaged.

Pearson’s correlation coefficient (*r*) and Spearman’s rank correlation coefficient (ρ) calculations were used to determine the strength of the relationships between parametric and non-parametric data sets, respectively, using NCSS/PASS Software (v. 2002, Kaysville, UT, USA). Significance was reached at *p* < 0.05. Spearman’s coefficients were calculated to compare the categorical data obtained from the functional scales with each other and with the sEMG-derived similarity index values. Comparisons were made between Non-injured and SCI groups for Magnitude and Similarity Index values using unpaired two-tailed *t*-test for samples of unequal variance. This calculation was performed for two sets of such data, one with all values and one with zero values removed to better show the relationship of incomplete lesions to the non-injured sEMG pattern.

## Results

### Clinical scale scores

The AIS category positively correlated with WISCI, SCIM Mobility, and SCIM Total scores while injury level correlated negatively with WISCI and SCIM Mobility scores (Table 2). FIM motor scores significantly correlated with WISCI and SCIM Self care, Mobility, and Total values. In addition, WISCI values correlated with SCIM Mobility and Total values.

### Motor control parameter values

**Figure 1:**
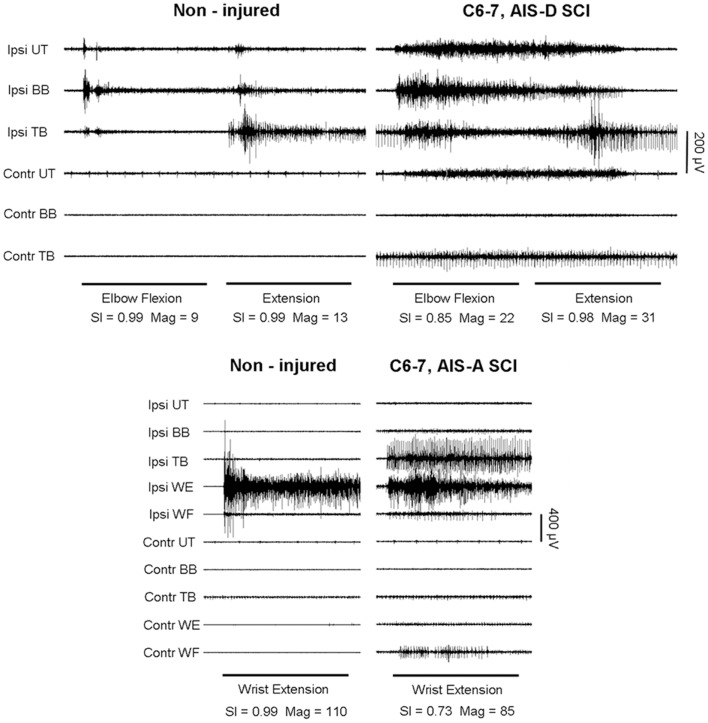
**Voluntary elbow flexion and extension (top) and wrist extension (bottom) in a representative non-injured subject and a person with motor-incomplete SCI (Subject #4)**. Muscles shown are Ipsilateral (Ipsi) and contralateral (Contr) upper trapezius (UT), biceps brachi (BB), triceps brachi (TB), wrist extensors (WE), and wrist flexors (WF). Vector analysis of surface EMG produced magnitude (Mag) and similarity index (SI) values shown for each task. Horizontal bars denote the 5-s cuing tone and analysis window. Note the presence of antagonist muscle contractions and the greater degree of ipsilateral and contralateral muscle co-activation in the SCI subject patterns compared to those of the non-injured subject.

**Figure 2:**
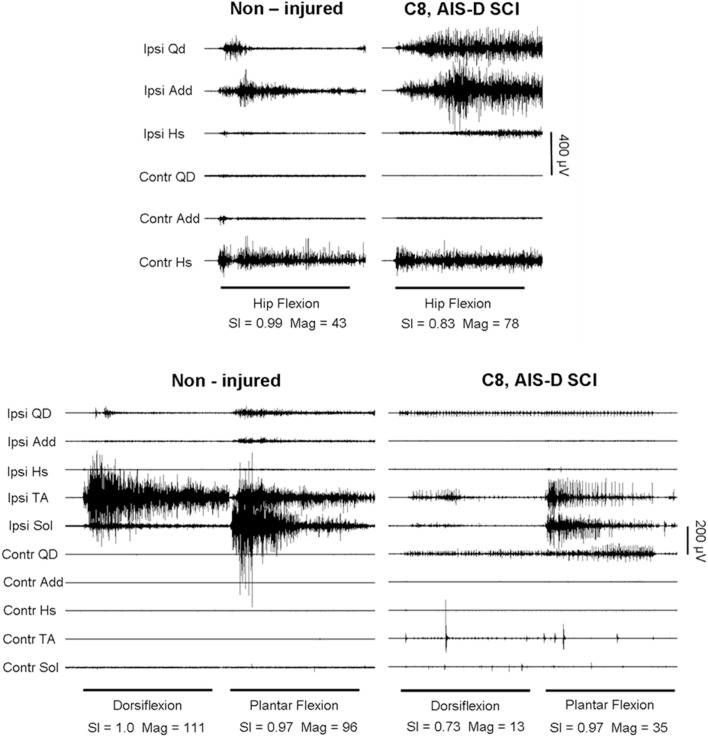
**Hip and knee flexion (top), ankle dorsiflexion and plantar flexion (bottom), in a non-injured subject and a person with motor-incomplete SCI (Subject #3)**. Muscles shown are Ipsilateral (Ipsi) and contralateral (Contr) quadriceps (Qd), adductor (Add), hamstring (Hs), tibialis anterior (TA), and soleus (Sol).

Compared to the multi-muscle patterns from healthy (non-injured) subjects, the reduced agonist muscle activation and excessive co-activation of antagonistic and other muscles significantly lowered SCI group Magnitude and SI values (Figures 1 and 2; Table 3). In those subjects who were able to produce measurable sEMG activity, group values were significantly lower for all but ankle dorsiflexion. The eight SCI subjects who were able to activate muscles during the ankle movement tasks produced SI and Magnitude values that, as a subgroup, were not significantly different from non-injured individuals. However, in this subgroup with non-responders removed, SI values for all upper limb tasks were significantly lower than those of the non-injured group.

**Table 3:** **Group similarity index and magnitude values for non-injured (*n* = 5) and SCI (*n* = 13) examinations**.

Voluntary task	Similarity index				Magnitude (μV)	
	Non-injured		SCI		Non-injured	SCI
	*n*	Mean ± SD	*n*	Mean ± SD	Mean ± SD	Mean ± SD
Elbow flexion	10	0.99 ± 0.02	13	0.68 ± 0.34**	60 ± 56	59 ± 45
Elbow extension		0.97 ± 0.03		0.66 ± 0.29**	18 ± 10	20 ± 16
Wrist extension		1.00 ± 0.00		0.75 ± 0.33*	207 ± 115	56 ± 40**
Hip and knee flexion	10	0.95 ± 0.03	26	0.54 ± 0.44**	45 ± 13	45 ± 42
Ankle dorsiflexion		1.00 ± 0.00		0.58 ± 0.47**	152 ± 58	78 ± 98**
Ankle plantar flexion		0.93 ± 0.06		0.55 ± 0.45**	77 ± 23	35 ± 38**

SCI group calculations made with and without zero values included. *p < 0.05; **p < 0.01.

### Motor control and clinical function scale scores

ASIA impairment scale grades for the nine studies in which both upper and lower limbs were assessed neurophysiologically, seven were judged to be D and one each were A and C precluding comparisons on the basis of AIS category. Also, no relationship was found between AIS per-muscle motor scores with the neurophysiological parameters, SI, and Magnitude. However, motor scores of 0 were always associated with no sEMG for the agonist of the motor task within this data set. Otherwise, no relationship was found between agonist muscle sEMG amplitudes and AIS motor scores.

**Figure 3:**
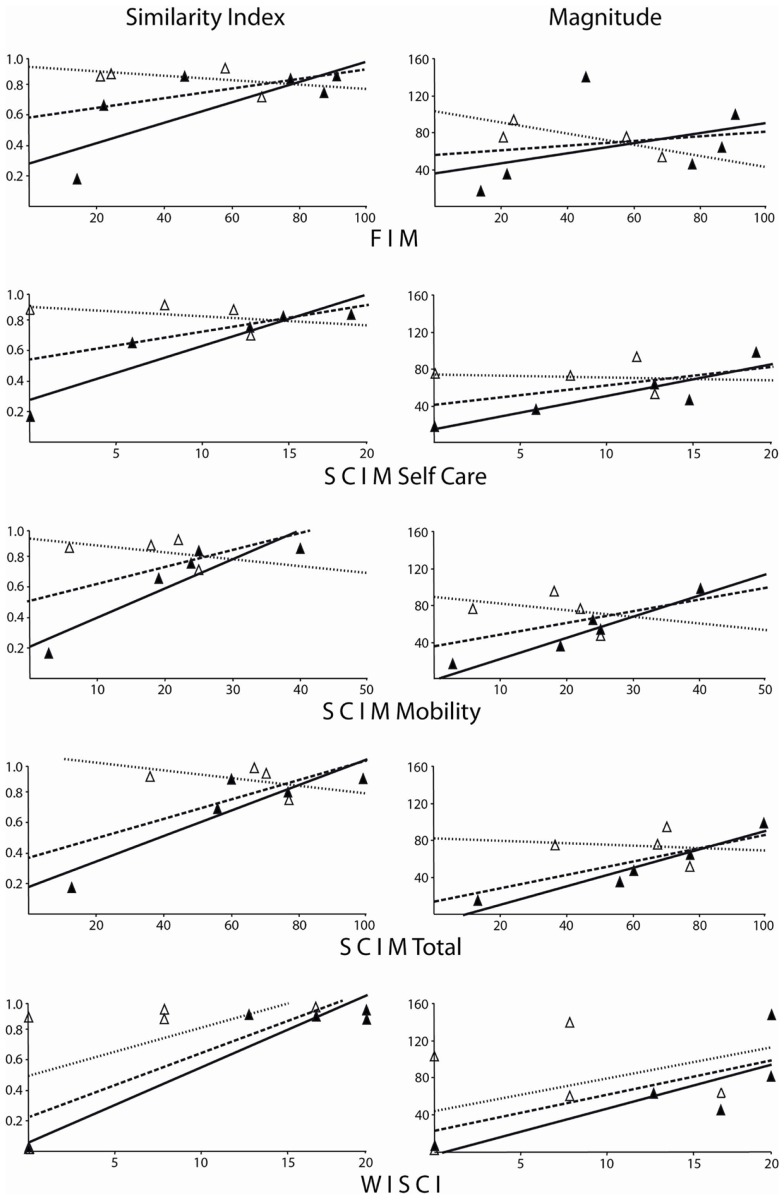
**Similarity and magnitude values corresponding to functional scale scores for FIM, SCIM, and WISCI examinations**. Open triangles are for early assessment post-injury and filled triangles are for late examinations. Linear trend lines are shown for overall (dashed), early (dotted), and late groupings (solid). Spearman’s correlation coefficients can be found in Table 4. For the overall, early and late combined grouping, only WISCI scale scores significantly correlated with neurophysiological parameters. SI and Mag values from late examination groupings correlated well with function scale values, reaching significance for SCIM and WISCI scales.

Spinal cord independence measure and WISCI scale values for assessments performed 48 or more days after injury were strongly and significantly correlated with SI and Mag values (Figure 3; Table 4). Correlation coefficients showed a non-significant relationship between neurophysiological parameters and late FIM, overall WISCI, and early WISCI scale values. One subject (#5) examined only 5 days after injury was scored as 0 on the WISCI but was able to perform lower limb tasks fairly well, serving as a clear example of how the early post-injury period may not be best assessed using function scales due to confounding factors limiting mobility.

**Table 4:** **Correlation values comparing clinical scale scores to neurophysiological sEMG-based magnitude (Mag) and similarity index (SI) overall, early (within 19 days) and late (after 48 days) post-injury**.

Correlation of function scales to motor control	Overall			Early post-injury			Late post-injury		
	*n*	SI ρ	Mag ρ	*n*	SI ρ	Mag ρ	*n*	SI ρ	Mag ρ
FIM motor	9	0.13	0.28	4	− 0.30	−0.80	5	0.70	0.66
SCIM self care	9	0.14	0.34	4	− 0.40	−0.40	5	1.00**	0.90*
SCIM mobility	9	0.13	0.26	4	− 0.20	−0.80	5	1.00**	0.90*
SCIM total	9	0.26	0.58	4	− 0.40	−0.40	5	0.90*	1.00**

		SI *r*	Mag *r*		SI *r*	Mag *r*		SI *r*	Mag *r*

WISCI	13	0.78	0.60	6	0.66	0.34	7	0.97**	0.87**

*p < 0.05; **p < 0.01.

In the two subjects who were examined twice, 1.5 and 6 months and 9 days and 5 months post onset, AIS category improved from C to D and injury level moved rostrally from C4 to C2 in the first and the AIS category remained A and the injury level moved rostrally from T8 to T5, respectively. Function scale scores and neurophysiological parameters increased between the two sessions in the first but remained at 0 in the person with the AIS-A categorization.

## Discussion

The method used here of calculating an index relating the spinal motor output of a test-subject to that of non-injured control subjects described the relationship between the quality of motor control and scores of commonly used clinical function scales in people with SCI during the acute and sub-acute phases of recovery. The primary finding was that the ability to produce spinal motor output with similar distribution and amplitude to non-injured subjects correlated well with clinical scale scores acquired more than 48 days after injury. However, such correlations were not found for examinations of clinical function acquired during the first 19 days post-injury.

To date, the AIS is the only assessment tool that has been recognized as a valid measure of the body functions and structures domains of the ICF for use in persons with SCI (3). AIS intra-rater reliability has been shown to be in the range of 80% with greatest agreement for motor scores of “0” and “5” and weakest for “3” (15) suggesting that across examiners, intermediate scores might induce a greater degree of variance. Multi-rater and multicenter reliability of the AIS was judged to be adequate for complete SCI but stability and sensitivity to change over serial evaluations in incomplete SCI subjects needed further study (2). Further, AIS sensitivity to minimal change and its ability to resolve meaningful change varied with SCI level and severity (16). From this, it might be surmised that the more diverse the lesion severity within a group, the less reliable will be the AIS tool. In the data presented here, seven of the nine studies in which all limbs were neurophysiologically assessed were from subjects judged to be AIS-D. With no AIS-C subjects, the previously published sensitivity of the neurophysiological measure to the degree of lesion severity as determined by the AIS (13) could not be repeated.

In a multicenter study that tracked recovery for 1 year in 160 people with SCI, MMT scores, and peak power output measured by hand-held dynamometry were found to positively correlate with FIM motor scores (17). However, in the current study, AIS-determined severity category and injury level failed to correlate with FIM motor scores. This could well be due, at least in part, to a limitation of the current study, the poor temporal synchronization of the AIS examination with other assessments used. Also, AIS category and level did not correlate with any of the neurophysiological parameter values. Correlation coefficient values comparing FIM to SI and Mag indicated a relationship within the late assessment grouping only but variability within the FIM data prevented it from being considered significant.

Clinical function measured by the SCIM-III, was determined in an international, evidence-based evaluation to provide the most appropriate overall description of functional ability in persons with SCI (18). SCIM-III self care scores correlated well with MMT scores and muscle strength was important to the performance of the functional tasks it measured (19). Further, in an international multicenter evaluation of 425 persons with SCI, agreement between raters using the SCIM-III was 75–96% for all tasks and results correlated significantly with FIM scores demonstrating its validity and reliability despite intercultural differences, and its superior sensitivity to changes in function compared with the FIM (20). In the current study, AIS category and injury level correlated significantly with the SCIM mobility sub-scale values. SCIM scores also correlated significantly with FIM motor scores and SI, and Magnitude values further supporting the notion that both the amount and organization of spinal motor output play a role in clinically relevant function after SCI. However, this was only true for the late assessment subgroup.

With regard to ambulation, the WISCI has been shown to provide good concurrent validity with FIM motor scores with high inter-rater reliability (10) and sensitivity to change (21). The AIS grade has shown some ability to predict ambulation after SCI with a greater percentage of AIS-C than AIS-A or B and more AIS-D than AIS-C graded individuals becoming ambulatory during their initial rehabilitation admission (22). The FIM was reported to have low sensitivity to locomotor and mobility issues (23). WISCI scores have also shown to correlate with SCIM lower extremity motor scores (24). Using a qualitative method to categorize multi-muscle sEMG activity during hip and knee flexion and extension in the supine position, it was possible to predict the degree of support and assistance needed for ambulation in a group with motor-incomplete SCI before the availability of the WISCI (25). In the current data, the sEMG-based SI and Magnitude parameters from lower limb tasks would have predicted the degree of support needed for ambulation based on strong correlations with WISCI scores but again, only within the late assessment subgroup.

The data presented here indicates that the relative distribution of spinal motor output to multiple muscles for simple, unloaded volitional tasks performed in the supine position is strongly related to clinically relevant function. Further, the neurophysiological approach used here avoids the sources of variance inherent in expert-examiner administered scales. Internal consistency across three trials of each task within the same recording session and test-retest consistency of the recorded sEMG patterns from which magnitude and SI are calculated was found to be high (8). Also, the use of vector-based analysis of the multichannel sEMG reduces the variance brought by skin and sub-dermal fat thickness, muscle size, and motor unit count within different muscles which vary across individuals and are known to impact sEMG amplitude-based measures.

The finding that the neurophysiological parameters presented here could be significantly correlated with FIM, SCIM, and WISCI scale scores suggests that the multi-muscle organization for voluntary movement may provide important additional information useful for evaluating and selecting intervention strategies and predicting functional recovery after SCI. These results also suggest that, at least for the period of hospitalization immediately after injury, the AIS and neurophysiological measures provide more accurate information about impairment and functional status than do function scale assessments due to confounding factors in the acute-care setting. Therefore, when patients are confined to their beds due to poly-trauma, post-surgical intervention, their need for respiratory support or other issues common to the acute period after injury, functional status may be better-determined using the neurophysiological method presented here. However, further longitudinal study is needed in a larger sample group is needed to examine and confirm the predictive capacity of this neurophysiological assessment approach.

## Conflict of Interest Statement

The authors declare that the research was conducted in the absence of any commercial or financial relationships that could be construed as a potential conflict of interest.
